# Complex Neurological Phenotype in Mutant Mice Lacking *Tsc2* in Excitatory Neurons of the Developing Forebrain^1,2,3^

**DOI:** 10.1523/ENEURO.0046-15.2015

**Published:** 2015-10-22

**Authors:** Beth Crowell, Gum Hwa Lee, Ina Nikolaeva, Valentina Dal Pozzo, Gabriella D’Arcangelo

**Affiliations:** 1 Department of Cell Biology and Neuroscience, Rutgers, The State University of New Jersey, Piscataway, New Jersey 08854; 2 Graduate Program in Molecular Biosciences, Rutgers, The State University of New Jersey, Piscataway, New Jersey 08854; 3 Graduate Program in Neuroscience, Rutgers, The State University of New Jersey, Piscataway, New Jersey 08854; 4 College of Pharmacy, Chosun University, Gwangju 501-759, South Korea

**Keywords:** astrocyte, mTOR, neuron, rapamycin, TSC, tuberous sclerosis complex

## Abstract

Mutations in the *TSC1* and *TSC2* genes cause tuberous sclerosis complex (TSC), a genetic disease often associated with epilepsy, intellectual disability, and autism, and characterized by the presence of anatomical malformations in the brain as well as tumors in other organs. The TSC1 and TSC2 proteins form a complex that inhibits mammalian target of rapamycin complex 1 (mTORC1) signaling. Previous animal studies demonstrated that Tsc1 or Tsc2 loss of function in the developing brain affects the intrinsic development of neural progenitor cells, neurons, or glia. However, the interplay between different cellular elements during brain development was not previously investigated. In this study, we generated a novel mutant mouse line (NEX-*Tsc2*) in which the *Tsc2* gene is deleted specifically in postmitotic excitatory neurons of the developing forebrain. Homozygous mutant mice failed to thrive and died prematurely, whereas heterozygous mice appeared normal. Mutant mice exhibited distinct neuroanatomical abnormalities, including malpositioning of selected neuronal populations, neuronal hypertrophy, and cortical astrogliosis. Intrinsic neuronal defects correlated with increased mTORC1 signaling, whereas astrogliosis did not result from altered intrinsic signaling, since these cells were not directly affected by the gene knockout strategy. All neuronal and non-neuronal abnormalities were suppressed by continuous postnatal treatment with the mTORC1 inhibitor RAD001. The data suggest that the loss of Tsc2 and mTORC1 signaling activation in excitatory neurons not only disrupts their intrinsic development, but also disrupts the development of cortical astrocytes, likely through the mTORC1-dependent expression of abnormal signaling proteins. This work thus provides new insights into cell-autonomous and non-cell-autonomous functions of Tsc2 in brain development.

## Significance Statement

The Tsc2 protein is defective in most cases of tuberous sclerosis complex (TSC), a disease that causes neurological symptoms, and is associated with brain malformations and tumors. Excitatory neurons are the most abundant cell type in the developing embryonic and perinatal brain. To understand how the loss of Tsc2 in these neurons affects overall brain development, we generated and characterized a novel conditional mouse mutant line that lacks Tsc2 specifically in developing excitatory neurons of the forebrain. We found that their intrinsic development as well as the development of other cell types such as astroglia is abnormal in mutant mice, suggesting that Tsc2 mutant neurons secrete extracellular signals that disrupt the development of the brain by affecting multiple cell types.

## Introduction

Tuberous sclerosis complex (TSC) is a genetic disorder caused by heterozygous inactivating mutations of either the *TSC1* or *TSC2* gene (for review, see Sahin, 2012; Crino, 2013). The disease is characterized by the presence of hamartomas and tumors in multiple organs, including the brain, and by neurological symptoms such as epilepsy, autism, and intellectual disability that affect 90–95% of the patients. Pathologically, TSC lesions in the brain include cortical tubers, subependymal nodules, and subependymal giant astrocytomas. Cortical tubers occur in most TSC patients; they are focal malformations that contain large dysmorphic neurons, giant neuroglial cells, and a variable number of astrocytes (Wong and Crino, 2012). Loss of *TSC1/TSC2* expression or function is thought to underlie the development of cortical tubers and tumors in individuals with TSC, whereas heterozygous levels of these genes affect neuronal connectivity and function without affecting brain structures, and predispose cells to loss of function (Tsai and Sahin, 2011).

The molecular activity of the TSC gene products has been partially elucidated (for review, see (Huang and Manning, 2008; Crino, 2011). The *TSC2* gene encodes a protein (Tsc2, tuberin) that contains a GTPase-activating protein (GAP) domain. The *TSC1* gene encodes a protein (Tsc1, Hamartin) that binds and stabilizes Tsc2. The Tsc1/Tsc2 complex inhibits the activity of Rheb via the GAP function of Tsc2. Since active Rheb positively regulates mammalian target of rapamycin complex 1 (mTORC1), a kinase complex that promotes protein synthesis, and increases cellular metabolism, the loss of Tsc1/2 results in widespread mTORC1 activation, resulting in cellular hypertrophy or increased proliferation. Decreased Tsc1/2 expression, as seen in heterozygous *Tsc1* mice, results in mTORC1 activation that is detectable only in enriched synaptic fractions, but not in whole-cell lysates (Bartley et al., 2014), and does not affect cell growth. The activity of Tsc2 is regulated and inhibited by Akt, a kinase that is critically involved in the control of cell growth. By inhibiting Tsc2, Akt strongly activates mTORC1 signaling. The loss of Tsc1/2 function, in turn, activates a feedback mechanism that inhibits Akt through inhibition of the upstream phosphoinositide 3 kinase (PI3K) pathway (Huang and Manning, 2009).

Several animal models have been generated to understand the role of the TSC proteins in cell and organ development. Constitutive *Tsc1* or *Tsc2* homozygous mouse mutants are not viable, but heterozygous mice and rats have been characterized. These rodents do not exhibit brain abnormalities, but exhibit behavioral and synaptic defects that are ameliorated by the inhibition of mTORC1 activity (Goorden et al., 2007; Ehninger et al., 2008). Conditional *Tsc1* and *Tsc2* mouse mutants have also been generated. Deletion of these genes in neural progenitor cells, producing neurons and glia, result in brain hypertrophy and seizures (Way et al., 2009; Goto et al., 2011; Magri et al., 2011; Zhou et al., 2011; Fu and Ess, 2013). Astroglia-specific deletion of *Tsc1* or *Tsc2* resulted in increased glial proliferation accompanied by epilepsy and premature death (Zeng et al., 2008; Zeng et al., 2011). Neuron-specific Tsc1 and Tsc2 knock-out (KO) mice, driven by the Synapsin1-Cre transgene, also exhibited premature mortality and seizures, accompanied by neuronal hypertrophy and myelination defects (Meikle et al., 2007; Wang et al., 2007). Furthermore, *Tsc1* deletion specifically in GABAergic interneurons caused increased seizure susceptibility, but no obvious structural abnormalities (Fu et al., 2012). However, no study so far examined the consequences of *Tsc1* or *Tsc2* loss specifically in excitatory neurons, the most abundant cell type in the developing forebrain. In this study, we used the NEX promoter to drive Cre expression and induce *Tsc2* gene deletion specifically in early postmitotic excitatory neurons of the developing forebrain. This genetic manipulation altered not only the intrinsic development of excitatory neurons, but also altered the development of cortical astrocytes through non-cell-autonomous mechanisms, providing insights into the molecular events underlying abnormal neuron–glia communication in TSC.

## Materials and Methods

### Animal handling and drug treatment

All animal procedures were performed in accordance with the regulations of the Animal Care Committee of Rutgers, The State University of New Jersey. Mice of either sex were used for this study. To generate the NEX-*Tsc2* mouse line, homozygous NEX-Cre knock-in mice (provided by Dr. Klaus Nave, Max Planck Institute, Göttingen, Germany; Goebbels et al., 2005) were crossed with loxP flanked *Tsc2* mice (*Tsc2* ^flox/flox^; Hernandez et al., 2007). NEX-Cre^+^;*Tsc2* ^flox/wt^ mice were interbred to generate wild-type [WT; NEX-Cre^+^;*Tsc2* ^wt/wt^, heterozygous (HT, NEX-Cre^+^;*Tsc2* ^wt/flox^), or homozygous mutant (KO; NEX-Cre^+^;*Tsc2* ^flox/flox^) mice]. tdTomato reporter mice (B6.Cg-*Gt(ROSA)26Sor^tm9(CAG-tdTomato)Hze^*/J) were obtained from The Jackson Laboratory. For RAD001 treatment, pharmaceutical grade RAD001 (>99% purity) was purchased from Selleck and dissolved in sterile DMSO to prepare a stock of 25 mg/ml. The stock was diluted 1:50 in PBS containing 0.5% Tween 80 (0.25 mg/ml). Pups were weighted and injected daily at the concentration of 10 mg/kg intraperitoneally starting at postnatal day 1 (P1). For most experiments, the last treatment was administered at P9, and the mice were killed at P10 for analysis. For one litter, the treatment was continued daily up to P30, and the mice were kept under observation up to P46.

### Tissue histology

Embryonic or newborn mice were killed at embryonic day 14 (E14) or P0, and the whole brain was dissected and immersed in perfusion solution containing 4% paraformaldehyde (PFA) in PBS. Older pups (P10) were perfused transcardially with 4% PFA in PBS and killed under anesthesia. These mice were anesthetized with Avertin (2,2,2-tribromoethanol dissolved in a tertiary amyl alcohol stock diluted in PBS), and perfused transcardially with PBS, pH 7.4, followed by 4% PFA in PBS. Dissected brains were post-fixed in 4% PFA in PBS overnight at 4°C and cryoprotected in 30% sucrose in PBS at 4°C. Brains were mounted onto a sliding microtome using OCT (Tissue-Tek) and sectioned in the sagittal or coronal plane at the 30 or 50 µm thickness. For histological analysis, thionin staining (FD Neurotechnologies) was performed according to the protocol of the manufacturer. In brief, sections were incubated in xylene for 3 min and then placed in 100% ethanol for 3 more minutes two times. Then, sections were incubated in 95% ethanol followed by 75% ethanol for 3 min each. Next, sections were placed in distilled water for 3 min three times, and processed for staining in a thionin solution for 10 min. Sections were briefly rinsed in distilled water and incubated in 95% ethanol 0.1% glacial acetic acid for 2 min. Finally, sections were dehydrated in 100% ethanol for 2 min (four times), cleared in xylene for 3 min (three times), and covered with Permount (Electron Microscopy Science) mounting medium.

### Immunostaining and immunofluorescence analysis

The sections were permeabilized with 0.1% Triton X-100 in PBS and blocked with 5% normal goat serum in 0.1% Triton X-100/PBS for 1 h at room temperature. The sections were incubated with primary antibodies overnight at 4ºC, followed by incubation with secondary antibodies for 1 h at room temperature. After washing in PBS for 5 min (three times), sections were mounted with Vectashield (Vector Laboratories) and imaged by confocal microscopy using a Yokogawa CSU-10 spinning disk attached to an inverted fluorescence microscope (model IX50, Olympus). The primary antibodies used in this study are as follows: rabbit anti-Tbr1 (Abcam), rabbit anti-Cux1 (Santa Cruz Biotechnology), goat anti-connective tissue growth factor (CTGF; L20, Santa Cruz Biotechnology), mouse anti-glial fibrillary acidic protein (GFAP; Cell Signaling Technology), rabbit anti-GFAP (Dako), rabbit anti-phospho-S6 (Ser235/236) ribosomal protein conjugated with Alexa Fluor 488 (Cell Signaling Technology), mouse anti-MAP2 (Covance), rabbit anti-Tau (Abcam), mouse anti-GAD67 (MAB5406, Millipore), and mouse anti-Olig2 (Millipore). Secondary antibodies were conjugated with Alexa Fluor 488 or Alexa Fluor 647 (Life Technologies). RedDot 2 (Biotium) or DAPI was used for nuclear counterstaining. To measure levels of phospho-S6 *in vitro*, corrected total cell fluorescence (CTCF) was calculated, using ImageJ to measure integrated density, mean gray value, and area of measurement. To generate a plot of GFAP signal intensity across the neocortex (from layer I to the white matter), the Plot Profile module of ImageJ was used. Intensity values were corrected by the subtraction of background and were averaged to the same number of intervals across the neocortex per section.

### Western blot analysis

The forebrain (neocortex and hippocampus) was dissected from P0 mice, whereas the neocortex and cerebellum from P10 mice were dissected. The tissues were lysed in RIPA buffer (50 mm Tris, pH 7.4, 1% NP40, 0.25% deoxycholate, 150 mm NaCl, 1 mm EGTA) supplemented with protease inhibitor (cOmplete, Roche) and phosphatase inhibitor (PhosSTOP, Roche), and cleared by centrifugation at 3000 × *g* for 5 min at 4°C. Protein lysates were loaded onto 8% or 10% SDS-PAGE gels, separated at 130 V for 2 h, and transferred to 0.22 µm nitrocellulose membranes. The membranes were blocked in 3% milk in 1 × TBS-T (Tris-buffered saline with 0.1% Tween 20) for 1 h at room temperature and incubated with primary antibodies overnight at 4°C. Membranes were incubated with secondary antibodies for 1 h at room temperature, washed with TBS-T for 5 min (3 times), and developed with the ECL-Plus Western Blotting Detection System (GE Healthcare) and autoradiographic film (Denville). The primary antibodies used in this experiment were as follows: rabbit anti-Tsc2 (Cell Signaling Technology), rabbit phospho-Akt Ser473 (Cell Signaling Technology), rabbit phospho-Akt Thr 308 (Cell Signaling Technology), rabbit Akt (Cell Signaling Technology), rabbit anti-PTEN (Cell Signaling Technology), rabbit anti-phospho-mTOR (Cell Signaling Technology), rabbit anti-mTor (Cell Signaling Technology), rabbit anti-phospho-S6 (Ser235/236) ribosomal protein (Cell Signaling Technology), mouse anti-S6 (Cell Signaling), rabbit anti-phospho-Erk1/2 (Thr202/Tyr204; Cell Signaling Technology), rabbit anti-total Erk1/2 (Cell Signaling Technology), mouse anti-β-actin-HRP (Sigma-Aldrich), mouse anti-Map2 (Covance), and mouse anti-GFAP (Cell Signaling Technology). Secondary antibodies were HRP conjugated (Sigma).

### Neuronal cultures

Hippocampal neuronal cultures were generated from P0 NEX-*Tsc2* littermates using a papain dissociation kit (Worthington). Cells were strained with a 70 µm filter, and centrifuged for 5 min at 300 × *g*. The pellet was resuspended in a solution of Earle’s balanced salt solution/ovomucoid protease inhibitor. The resuspension was layered on top of 5 ml of ovomucoid protease inhibitor solution, centrifuged for 6 min at 200 × *g*, and then centrifuged for an additional 6 min at 300 × *g* to evenly coat the cells with the ovomucoid solution. Cells were resuspended in a mixture of 98% Neurobasal medium, 2% B-27 supplement, 0.5 mm glutamine, and 0.5 mm Penn/Strep, plated onto poly-l-lysine-coated glass coverslips at a density of 50,000 cells/cm^2^, and maintained at 37°C in 5% CO_2_ in a water-jacked incubator for 10 days *in vitro* (DIV). At DIV10, cells were washed twice with PBS and fixed for 20 min in 4% paraformaldehyde in PBS at room temperature. Cells were then permeabilized for 10 min in 0.1% Triton-X in PBS, washed, and blocked in 10% normal goat serum prior to immunofluorescence staining. Levels of immunofluorescence were calculated from confocal images of *n* > 145 neurons/genotype after subtracting the background and calculating the CTCF by ImageJ.

### Statistical analysis

Statistical analysis was conducted using the GraphPad Prism software. Western blot data shown in bar graphs represent the mean ± SEM and were analyzed using Kruskal–Wallis test. The Kolmogorov–Smirnov test was used to compare the distribution of the GFAP fluorescence intensity between two genotypes. Expression levels of phospho-S6 *in vitro* were compared between two genotypes and analyzed using the Mann–Whitney *U* test. The results were averaged from multiple sections obtained from multiple animals. Statistical significance was determined when the *p* value was <0.05 (Table 1).

**Table 1.** Statistical analysis

	Data structure	Statistical test	Power
a	Normality not assumed	Kruskal–Wallis test	*p* = 0.0070
b	Normality not assumed	Kruskal–Wallis test	*p* = 0.0035
c	Normality not assumed	Kolmogorov–Smirnov test	*p* < 0.0001
d	Normality not assumed	Kruskal–Wallis test	*p* = 0.8521
e	Normality not assumed	Kruskal–Wallis test	*p* = 0.0240
f	Normality not assumed	Kruskal–Wallis test	*p* = 0.0090
g	Normality not assumed	Kruskal–Wallis test	*p* = 0.1767
h	Normality not assumed	Kruskal–Wallis test	*p* = 0.0275
i	Normality not assumed	Kruskal–Wallis test	*p* = 0.0132
j	Normality not assumed	Kruskal–Wallis test	*p* = 0.7934
k	Normality not assumed	Kruskal–Wallis test	*p* = 0.9565
l	Normality not assumed	Kruskal–Wallis test	*p* = 0.0753
m	Normality not assumed	Kruskal–Wallis test	*p* = 0.3157
n	Normality not assumed	Kruskal–Wallis test	*p* = 0.0390
o	Normality not assumed	Kruskal–Wallis test	*p* = 0.3932
p	Normality not assumed	Mann–Whitney test	*p* < 0.0001
q	Normality not assumed	Mann–Whitney test	*p* = 0.5251
r	Normality not assumed	Mann–Whitney test	*p* = 0.9075
s	Normality not assumed	Mann–Whitney test	*p* < 0.0001
t	Normality not assumed	Mann–Whitney test	*p* = 0.7454 at 12.5 µm *p* = 0.0046 at 25.0 µm *p* = 0.0139 at 37.5 µm *p* = 0.0290 at 50.0 µm *p* = 0.2292 at 62.5 µm *p* = 0.1862 at 75.0 µm *p* = 0.2434 at 87.5 µm *p* = 0.3587 at 100.0 µm *p* = 0.0745 at 112.5 µm
u	Normality not assumed	Kruskal–Wallis test	*p* < 0.0001
v	Normality not assumed	Kruskal–Wallis test	*p* = 0.0002
w	Normality not assumed	Kruskal–Wallis test	*p* = 0.9946

#### Results

### Generation and neuroanatomical characterization of forebrain excitatory neuron-specific *Tsc2* knock-out mice

**Figure 1.**
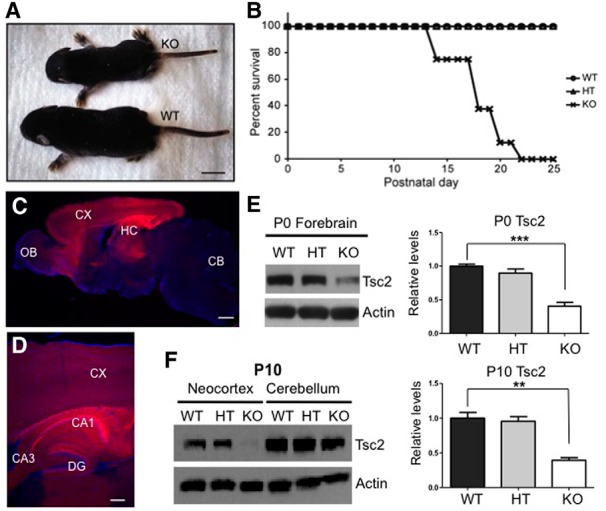
Phenotype of NEX-*Tsc2* mutant mice and forebrain-specific gene deletion. ***A***, Appearance of WT and KO NEX-*Tsc2* littermates at P9. The KO mouse appears runt. ***B***, Cumulative survival curve of cohorts of WT, HT, and KO NEX-*Tsc2* littermates reared in the same cages. ***C***, ***D***, Representative brain images obtained from a P10 NEX-Cre^+^ mouse expressing the tdTomato reporter gene at 4× (***C***) or 10× magnification (***D***). The images show predominant reporter gene expression in the forebrain, with elevated activity in the cerebral cortex, hippocampal area CA1, and dentate gyrus. ***E***, ***F***, Western blot analysis and quantification of Tsc2 in selected brain regions of NEX-*Tsc2* littermates at P0 (***E***) and P10 (***F***). Blots were reprobed with antibodies against actin as a loading control. Tsc2 levels were significantly reduced in KO forebrain regions, but not in the cerebellum. Bar graphs show the mean values relative to WT, ±SEM; **p < 0.01; ***p < 0.001. CX, Cerebral cortex; HC, hippocampus; CB, cerebellum; OB, olfactory bulb; DG, dentate gyrus, Scale bars: ***A***, 1 cm; ***C***, 500 µm; and ***D***, 200 µm.

To explore the effects of the *Tsc2* gene loss in excitatory neurons of the developing forebrain, we generated a conditional KO mouse line in which *Tsc2* gene deletion is driven by Cre expression under the NEX promoter (NEX-*Tsc2*). This promoter is active specifically in early postmitotic excitatory neurons of the embryonic forebrain, but not in dividing radial progenitor cells, glial cells, or inhibitor neurons (Goebbels et al., 2005; Belvindrah et al., 2007). Our conditional NEX-*Tsc2* line was generated by crossing NEX-Cre^+^ knock-in mice with *Tsc2* ^flox/flox^ mice (Hernandez et al., 2007), and then interbreeding double heterozygous Cre^+^;*Tsc2* ^flox/wt^ mice. This breeding scheme generated Cre^+^;*Tsc2* ^flox/flox^ homozygous mutants (KO), heterozygous Cre^+^;*Tsc2* ^wt/flox^ (HT), and WT controls that were either Cre^+^;*Tsc2* ^wt/wt^ or Cre^−^. NEX-*Tsc2* KO mice were born according to a mendelian ratio and appeared indistinguishable from control littermates. However, beginning at ∼P5, they failed to gain weight at the same rate as littermate controls and appeared increasingly runt (Fig. 1*A*). By P10, the body weight of KO mice was significantly reduced compared with controls (69.6 ± 6.3%; Kruskal–Wallis test, *p* < 0.001^v^; Table 2), although the brain weight was similar across genotypes (Kruskal–Wallis test, *p* > 0.05^w^; Table 2). KO mutant mice exhibited premature mortality, and many began to die at ∼P12–P14. The median survival age for KO mice was P16, and no mutant mice survived past P22 (Fig. 1*B*). Unlike KO mice, HT mice appeared unaffected and were indistinguishable from WT controls. No overt seizure activity was noted at any time in HT or KO mice.

**Table 2.** Body and brain weight of NEX-*Tsc2* mice at P0 and P10

Genotype	P0		P10	
	Body weight (g)	Brain weight (g)	Body weight (g)	Brain weight (g)
WT	1.48 ± 0.03 (*n* = 23)	0.12 ± 0.00 (*n* = 23)	5.74 ± 0.11 (*n* = 23)	0.36 ± 0.00 (*n* = 22)
HT	1.38 ± 0.07 (*n* = 4)	0.12 ± 0.01 (*n* = 4)	6.14 ± 0.2 (*n* = 13)	0.36 ± 0.01 (*n* = 10)
KO	1.36 ± 0.06 (*n* = 5)	0.12 ± 0.01 (*n* = 5)	4.00 ± 0.36* (*n* = 7)	0.36 ± 0.01 (*n* = 5)
WT + RAD001	ND	ND	3.03 ± 0.34 (*n* = 4)	0.19 ± 0.01 (*n* = 4)
HT + RAD001	ND	ND	3.27 ± 0.09 (*n* = 10)	0.21 ± 0.00 (*n* = 10)
KO + RAD001	ND	ND	2.80 ± 0.26 (*n* = 3)	0.21 ± 0.00 (*n* = 3)
				

Values indicate the average weight ± SEM. ND, Not determined.

*Statistically significant difference between KO and WT mice in the untreated group (Kruskal–Wallis test, *p* < 0.001^p^).

Given the premature mortality of KO mice, in this study we limited our analysis to mice up to the age of P10. To confirm the regional specificity of the *Tsc2* gene loss at postnatal ages, we first crossed NEX-Cre^+^ knock-in mice with transgenic tdTomato reporter mice and examined the expression of the fluorescent reporter protein in the progeny by confocal microscopy. At P10, the brain of Cre^+^;tdTomato^+^ mice displayed an intense red fluorescence signal that was predominantly localized to forebrain structures such as the cerebral cortex and the hippocampus (Fig. 1*C*,*D*). Some reporter expression was also noted in few cells of the midbrain and the hindbrain. The fluorescence signal was intense in all cellular layers and in the developing white matter of the neocortex. However, in the hippocampus the signal was restricted mostly to the pyramidal cell layer and the neuropil in area CA1, and to mature granule cells in the outer granular layer of the dentate gyrus and to their processes, including axonal projections into hippocampal area CA3 (Fig. 1*D*). Pyramidal cells in area CA3 did not exhibit reporter expression. This expression pattern is remarkably similar to that of reporter genes expressed from the CamKIIα promoter at older ages (>P21; Tsien et al., 1996; Trotter et al., 2013) and confirms that the NEX-Cre driver line induces genetic recombination almost selectively in the forebrain at postnatal as well as embryonic ages. To verify region-specific loss of *Tsc2* expression in NEX-*Tsc2* KO mice, we conducted Western blot analysis of dissected brain regions at P0 and P10. The data show that the levels of Tsc2 protein were strongly and significantly reduced in the forebrain of KO mice at P0 (Kruskal–Wallis test, *p* < 0.01^a^; Fig. 1*E*). When P10 brain tissue was further dissected, Tsc2 levels were also significantly reduced in the neocortex of KO mice (Kruskal–Wallis test, *p* < 0.01^b^), but not in the cerebellum (Fig. 1*F*). However, the levels of Tsc2 protein did not appear to be significantly reduced in forebrain tissue of HT mice at P0 or P10, despite the loss of one allele. This is likely due to the residual expression of Tsc2 in brain cells other than excitatory neurons.

### Neuronal abnormalities in NEX-*Tsc2* mutant mice

**Figure 2.**
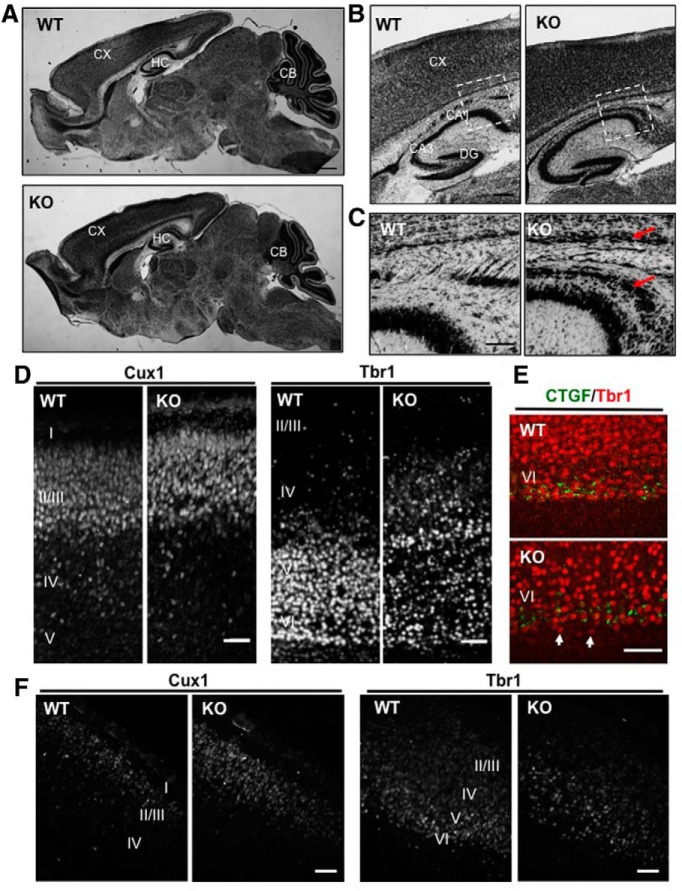
Anatomical analysis of forebrain structures in NEX-*Tsc2* mice. ***A–C***, Sagittal sections of P10 brain tissue from WT and KO mice were stained with thionin. ***A***, All main brain regions appear normal at low magnification. ***B***, ***C***, Further magnified images of the mutant forebrain reveal the presence of an abnormally thick cellular layer in the deep neocortex, and dispersed pyramidal cells in hippocampal area CA1 (arrows). ***D***, ***E***, Layer marker analysis in the P0 neocortex. Sagittal sections were processed for immunofluorescence with the indicated antibodies. Cux1-positive upper layer neurons formed a normal, compact cellular layer, whereas Tbr1-positive deep layer neurons were somewhat dispersed in the KO sections compared with WT sections, and were sometimes positioned below the normally placed subplate neurons identified by the CTGF antibody (arrows). ***F***, Layer marker analysis in the E14 neocortex. Coronal sections were processed for immunofluorescence with Cux1 or Tbr1 antibodies. Scale bars: ***A***, 500 µm; ***B***, 200 µm; ***C***, 100 µm; ***D–F***, 50 µm.

To analyze the neuroanatomy of NEX-*Tsc2* mice, we first stained brain sections of P0 and P10 KO, HT and WT animals with thionin. All major cortical structures of mutant mice appeared normal in size and overall architecture (Fig. 2*A*,*B*; data not shown). However, discrete structural abnormalities were noted in the forebrain of P10 KO mice. An abnormally thick bundle of cells was present in the deepest aspect of the neocortex, just above the white matter (Fig. 2*B*,*C*). Also, a dispersion of pyramidal cells in hippocampal area CA1 was consistently observed (Fig. 2*B*,*C*). No neuroanatomical defect was noted in HT NEX-*Tsc2* mice (data not shown).

To investigate in more detail the anatomy of the neocortex, we conducted immunofluorescence using layer-specific antibodies, such as Cux1, which labels principal neurons in upper cortical layers II/III, Tbr1, which labels principal neurons in deep layers V/VI, and CTGF, which labels subplate neurons. Cux1 and CTGF expression appeared normal in KO mice at P0 (Fig. 2*D*,*E*). The position of Tbr1-positive neurons was mostly normally restricted to deep cortical layers; however, this layer was not as compact in KO mice as in controls, and some Tbr1-expressing cells were abnormally dispersed in more superficial layers of the cortical plate or beneath the subplate (Fig. 2*D*,*E*). Similarly, results were seen in NEX-*Tsc2* mice at E14 (Fig. 2*F*) and P10 (data not shown), although layer marker expression was reduced overall, especially in postnatal KO sections. These results suggest that the loss of Tsc2 expression in the developing brain modestly disrupts neuronal migration, primarily affecting the positioning of early-born principal neurons destined for deep cortical layers.

### Glial abnormalities in NEX-*Tsc2* mutant mice

**Figure 3.**
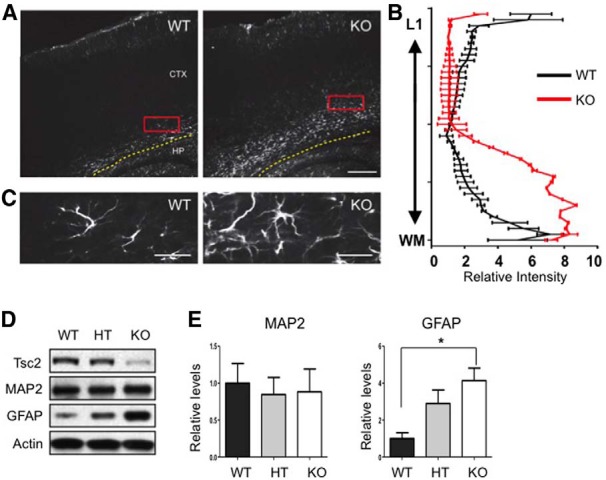
Increased astrogliosis in the NEX-*Tsc2* KO forebrain. ***A***, ***C***, Immunofluorescence analysis of GFAP expression in P10 brain sections revealed a wide distribution of GFAP-positive astrocytes in the neocortex of NEX-*Tsc2* KO mice. Images at low (***A***) and high magnification (***C***) show an increased number of astrocytes in the neocortex. ***B***, GFAP signal intensity plot in the neocortex in WT or KO NEX-*Tsc2* mice. Data quantified from multiple sections and *n* = 3 mice per genotype demonstrate a statistically significant increase in GFAP staining intensity in KO compared to WT mice in the deepest and most marginal layers of the neocortex (*p* < 0.0001, Kolmogorov–Smirnov test). ***D***, Western blot analysis of cortical lysates obtained from NEX-*Tsc2* mice at P10. GFAP expression was significantly increased in KO compared with WT mice, whereas MAP2 expression was normal. ***E***, Quantification of Western blot data obtained from *n* = 5 mice per genotype. Scale bars: ***A***, 200 µm; ***C***, 25 µm. Bar graphs show the mean values relative to WT, ±SEM; *p < 0.05.

Cortical tubers in TSC patients are characterized by the presence of hypertrophic, dysplastic neurons that are believed to experience complete loss of *TSC1* or *TSC2* function and mTORC1 activation. In addition, cortical tubers often exhibit an increased number of glial elements (Mizuguchi and Takashima, 2001; Sosunov et al., 2008; Wong and Crino, 2012), which could be the result of intrinsic *Tsc2* loss of function or, alternatively, could be the result of altered neuron–glia communication. Our NEX-*Tsc2* mice provided us with an opportunity to distinguish between these possibilities and determine whether glial cells are affected by the loss of *Tsc2* in postmitotic neurons. We first used an immunofluorescence assay to examine the distribution and morphology of astroglial elements labeled by the GFAP antibody. In the P10 WT neocortex, GFAP expression was mainly restricted to the marginal zone and to the white matter in the deep aspect of the cortex (Fig. 3*A*). In mutant cortex, in contrast, the thickness of GFAP-positive layers was noticeably increased, and astrocytes appeared to invade the cortical plate (Fig. 3*A*). The distribution of the GFAP signal in the neocortex was quantitatively analyzed by measuring the fluorescence intensity with the ImageJ/plot profile. Significant differences in GFAP intensity between WT and KO mice were observed in both, the most superficial and the deeper cortical layers (Fig. 3*B*; Kolmogorov–Smirnov test, *p* < 0.0001^c^). At higher magnification, it was also apparent that these cells exhibited a branched morphology typical of reactive astrocytes; however, they did not appear hypertrophic (Fig. 3*C*). No increase of GFAP expression was noted in the KO hippocampus (not shown). Western blot analysis was also used to confirm our findings in the P10 neocortex. The levels of the neuron marker MAP2 were unchanged; however, the mean GFAP levels were significantly elevated in KO mutants compared with WT controls, despite a considerable variability across individual animals of the same genotype (Kruskal–Wallis test, *n* = 5 mice, *p* < 0.05^d,e^; Fig. 3*D*,*E*). Interestingly, GFAP expression also appeared to be increased in HT mice compared with WT controls, although the values did not reach statistical significance. We also performed TUNEL staining of P10 sections to evaluate possible cell loss in conjunction with astrogliosis. Very few TUNEL-positive cells were detected in the forebrain of NEX-*Tsc2* mice; however, no difference was noted between WT and KO samples (data not shown), indicating that the loss of neuronal Tsc2 expression does not cause cell death, and that the observed astrogliosis is not due to cell death.

Our data indicate that at least one glial cell type, the cortical astrocyte, is significantly affected by the targeted deletion of the *Tsc2* gene in postmitotic neurons of the developing forebrain.

### Signaling abnormalities in the forebrain of NEX-*Tsc2* mice

**Figure 4.**
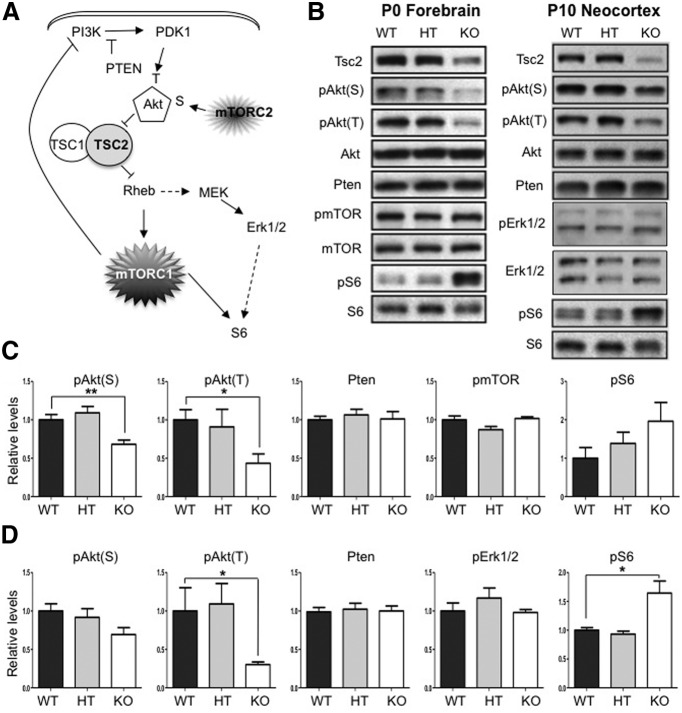
Analysis of PI3K/Akt/mTOR signaling in the neocortex in NEX-*Tsc2* mice. ***A***, Diagram of the PI3K/Akt/mTOR signaling pathway. ***B***, Western blot analysis of tissue lysates obtained from WT, HT, and KO NEX-*Tsc2* mice at P0 or P10. ***C***, Quantification of P0 Western blot data obtained from *n* = 3 mice per genotype. ***D***, Quantification of P10 Western blot data obtained from *n* = 4–5 mice per genotype. pAkt(T) and pAkt(S) levels were reduced, whereas pS6 levels were increased in KO mice compared with WT controls. Pten and pErk1/2 levels were unaffected. Bar graphs show the mean values relative to WT, ±SEM; *p < 0.05; **p < 0.01.

Given the well known impact of Tsc2 on the activity of the mTOR kinase, we investigated downstream targets of both the mTORC1 and mTORC2 complexes, and putative feedback mechanisms affecting the PI3K/Akt signaling pathway (Fig. 4*A*). We analyzed forebrain lysates obtained from P0 NEX-*Tsc2* mice and neocortical lysates obtained from P10 mice by Western blotting using antibodies against phosphorylated or total targets. The levels of phosphorylated proteins were normalized to corresponding protein total levels, whereas the levels of nonphosphorylated proteins were normalized to actin as a loading control. In both sets of samples, Tsc2 levels were significantly reduced in KO compared with WT mice, as expected, but did not differ in HT mice (Figs. 1*E*,*F*, 4*B*). Akt activity was probed using antibodies that recognize two different phosphorylation sites, serine 473 (the target of mTORC2) and threonine 389 (the target of the PI3K-dependent Pdk1 kinase). Akt phosphorylation at both sites was reduced in KO mice at both ages (Fig. 4*B*). However, phospho-serine 473 levels [pAkt(S)] were significantly reduced in P0 samples (Fig. 4*C*), but not in P10 samples (Kruskal–Wallis test, *p* < 0.01 and *p* > 0.05, respectively^f,g^; Fig. 4*D*), whereas phospho-threonine 308 levels [pAkt(T)] were significantly reduced in both sets of samples (Kruskal–Wallis test, *p* < 0.05^h,i^). To investigate whether decreased Akt phosphorylation on this PI3K-dependent site may result from increased Pten levels, we examined the expression of this phosphatase. However, Pten levels were unaffected in KO mice at both ages (Kruskal–Wallis test, *p* > 0.05^j,k^; Fig. 4*B–D*). Reduced Akt phosphorylation could also be due to the compensatory downregulation of PI3K activity that occurs in response to persistent activation of mTORC1 (Huang and Manning, 2009). mTORC1 activity is well known to be induced in response to Tsc1 or Tsc2 loss due to the increased function of the GTP-binding protein Rheb (Laplante and Sabatini, 2009). There was no significant change in mTOR phosphorylation at P0 (Kruskal–Wallis test, *p* > 0.05^l^). To investigate mTORC1 activity in NEX-*Tsc2* mice, we examined the phosphorylation levels of the ribosomal protein S6 (pS6). Phosphorylation of this protein is known to be mTORC1 dependent and is commonly used as a readout of mTORC1 activity (Meikle et al., 2007; Zeng et al., 2008; Ljungberg et al., 2009; Way et al., 2009). Indeed, we found that pS6 levels were elevated in KO mice compared with WT mice (Fig. 4*B*). However, values were not statistically significant at P0 (Kruskal–Wallis test, *p* > 0.05^m^; Fig. 4*C*), but were significant at P10 (Kruskal–Wallis test, *p* < 0.05^n^; Fig. 4*D*). Since MEK/Erk1/2 signaling potentially influences the phosphorylation levels of S6 in addition to mTORC1 (Fig. 4*A*), and Tsc1 loss has been recently shown to induce Erk1/2 activity in mutant mice (Zhang et al., 2014), we also examined the phosphorylation levels of Erk1/2 (pErk1/2) in the P10 neocortex of NEX-*Tsc2* mice. However, no changes were detected in KO samples (Kruskal–Wallis test, *p* > 0.05^°^; Fig. 4*B*,*D*), suggesting that the induction of pS6 levels are due to the activation of mTORC1 signaling. Consistent with the apparently normal levels of Tsc2, no Akt/mTOR signaling abnormalities were detected in HT NEX-*Tsc2* mice at any age analyzed (Fig. 4*B–D*).

Together, these data indicate that homozygous loss of the *Tsc2* gene in the forebrain leads to significant mTORC1 activation and downregulation of Akt activity, whereas heterozygous deletion has no detectable effects on Tsc2 protein levels and Tsc2-dependent signal transduction.

### Neuron-specific activation of mTORC1 signaling in NEX-*Tsc2* mutants

**Figure 5.**
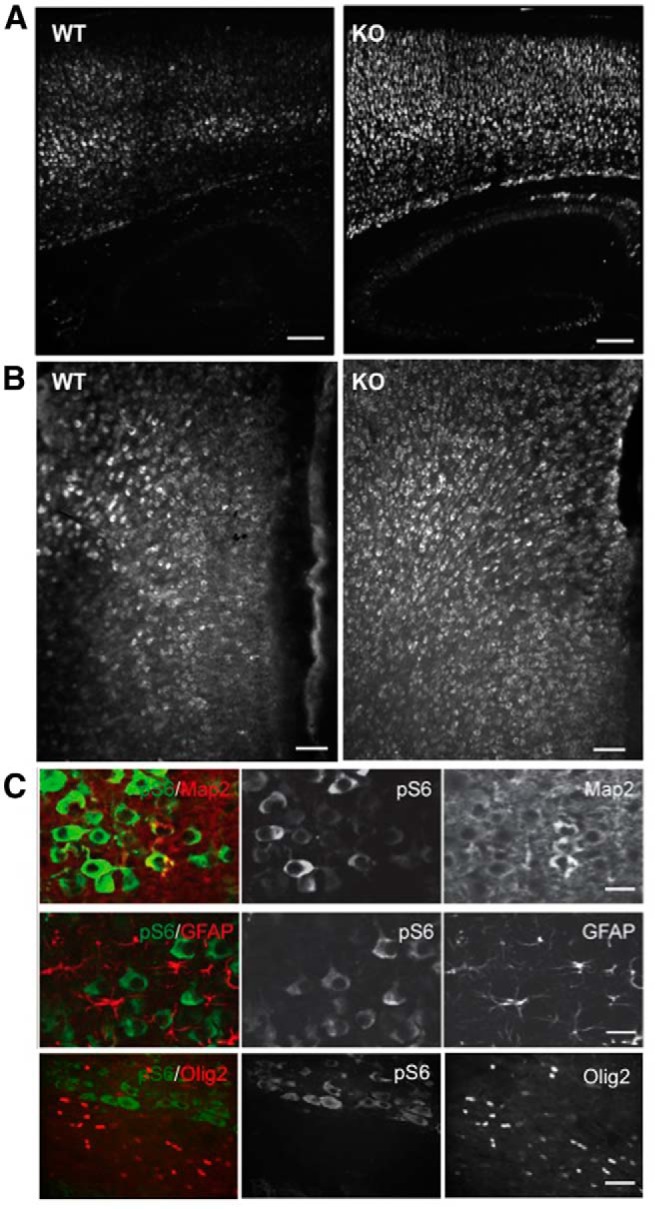
Neuron-specific activation of mTORC1 in the KO NEX-*Tsc2* forebrain at P10. ***A***, Low-magnification images of the somatosensory cortex and hippocampus obtained from sagittal sections of WT and KO NEX-*Tsc2* brains stained with pS6 antibodies. ***B***, Low-magnification images of the anterior cingulate cortex obtained from coronal sections of WT and KO NEX-*Tsc2* brains stained with pS6 antibodies. The pS6 signal is stronger in all areas of the KO forebrain compared with the WT. ***C***, High-magnification confocal images of KO neocortical sections double labeled with pS6, and the indicated cell marker antibodies show that the pS6 signal colocalizes with the neuronal marker Map2, but not with the astrocyte marker GFAP or the oligodendrocyte marker Olig2. Scale bars: ***A***, 200 µm; ***B***, 100 µm; ***C***, 20 µm.

To examine cell-specific activation of mTORC1 signaling, we conducted *in vivo* and *in vitro* double-immunofluorescence experiments using antibodies against the mTORC1 target pS6 and cell-specific markers. The intensity of the pS6 staining in the P10 KO neocortex was markedly increased compared to WT neocortex (Fig. 5*A*,*D*), which is consistent with our Western blotting results (Fig. 4). In sagittal sections through the somatosensory cortex, this staining was elevated in all cortical layers, and it was particularly evident in the abnormally thick deep cellular layer present in the mutant cortex (Fig. 5*A*). Similarly, strong pS6 staining was observed in coronal sections through the anterior cingulate cortex of KO mice, where positive cells were seen in all cellular layers (Fig. 5*B*). Double staining with MAP2 antibodies demonstrates that the great majority of intensely labeled pS6-positive cells of the KO neocortex were indeed neurons (Fig. 5*C*). These cells appear clearly enlarged compared to pS6-positive neurons in the WT cortex. On the other hand, double labeling with GFAP or Olig2 antibodies revealed a virtually complete lack of signal colocalization (Fig. 5*C*). These data indicate that mTORC1 is activated specifically in forebrain neurons but not in glial cells, which is consistent with the genetic design of NEX-*Tsc2* mice.

**Figure 6.**
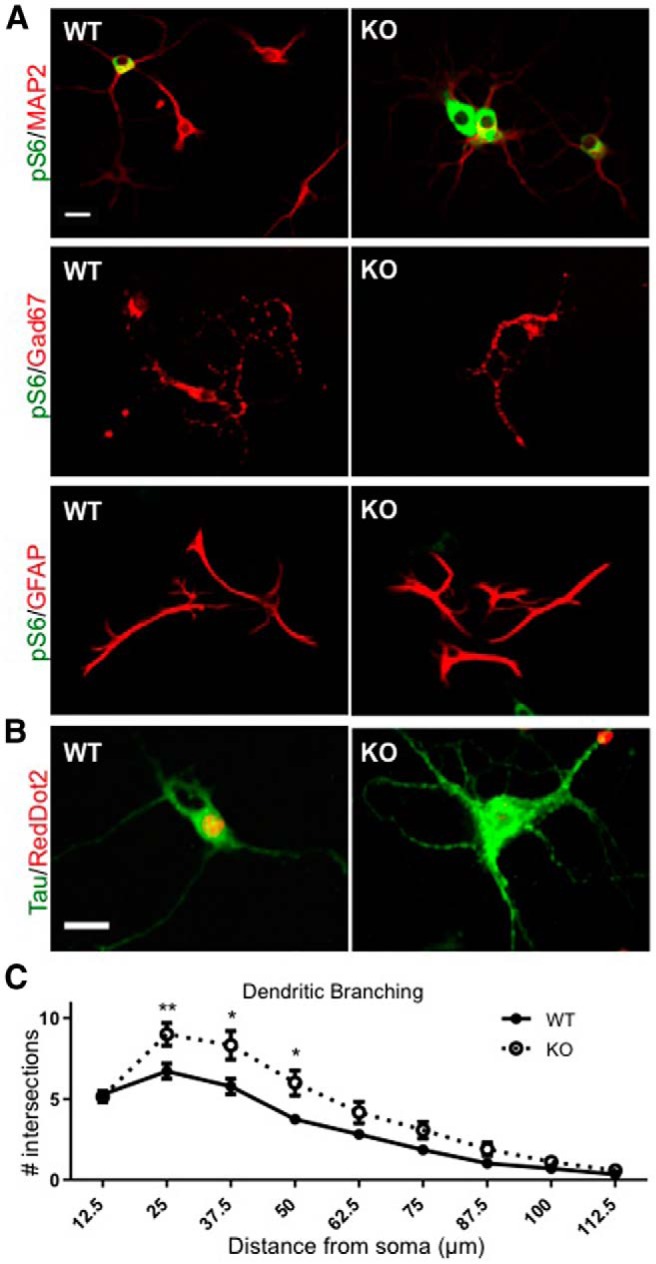
Analysis of WT and KO hippocampal neurons in culture. Neurons were dissociated from newborn mice and cultured for 10 DIV. ***A***, Double immunofluorescence with pS6 and the indicated antibodies demonstrates excitatory neuron-specific activation of mTORC1 in KO neurons. Strong pS6 signal was seen preferentially in KO cultures and was colocalized with the MAP2 signal in neurons, but not with the Gad67 signal in inhibitory neurons or the GFAP signal in astrocytes. Scale bar, 20 µm. ***B***, Neurons were double stained for the axon-specific marker Tau and the nuclear stain RedDot2. KO neurons exhibit an increased number of axons compared with WT neurons. Scale bar, 20 µm. ***C***, Sholl analysis of MAP2-positive dendrites in WT and KO hippocampal cultures. KO neurons showed increased dendritic branching within 50 µm of the soma, ±SEM; *p < 0.05; **p < 0.01.

To confirm these findings with a higher resolution, and to analyze the cellular processes of mutant neurons in more detail, we cultured NEX-*Tsc2* KO and WT hippocampal neurons for 10 DIV. Double labeling with pS6 and cell-specific markers unequivocally demonstrated that levels of pS6 were significantly elevated in MAP2-positive neurons of KO cultures compared with WT counterparts (*n* = 145-155 neurons/genotype; Mann–Whitney *U* test, *p* < 0.0001^p^; Fig. 6*A*). Average levels of pS6 in KO MAP2-positive neurons were increased by ∼10-fold compared with WT levels. Furthermore, Gad67-positive interneurons and GFAP-positive astrocytes in the mutant KO cultures exhibited no induction of pS6 levels compared with cells in WT cultures (*n* > 46 Gad67-positive and *n* > 54 GFAP-positive cells/genotype; Mann–Whitney *U* test, *p* > 0.05^q,r^; Fig. 6*A*). These data demonstrate that the deletion of *Tsc2* in excitatory neurons leads to cell-autonomous activation of mTORC1 signaling in these cells, but does not affect mTORC1 signaling in surrounding brain cell types. To examine the effect of increased mTORC1 signaling on the growth of cellular processes, we stained WT and KO hippocampal cultures with Tau antibodies to label axons, or MAP2 antibodies to label dendrites. KO neurons exhibited a significant increase in the number of axonal processes emanating from the soma compared with WT neurons (Fig. 6*B*; *n* > 37 neurons/genotype; Mann–Whitney *U* test, *p* > 0.0001^s^). The number of MAP2-positive dendritic branches also appeared increased in KO neurons compared with WT neurons (Fig. 6*A*). Sholl analysis demonstrated that KO neurons exhibit increased dendritic branching compared with WT neurons. Proximal dendrites were particularly affected, and statistical differences were measured within a distance of 50 µm from the soma (*n* > 40 neurons/genotype; Mann–Whitney *U* test, *p* > 0.05^t^; Fig. 6*C*). Together, these data demonstrate that the intrinsic loss of Tsc2 and mTORC1 activation causes overall neuronal hypertrophy that is characterized by an increase in multiple parameters, such as soma size, and axon and dendrite branching.

### mTORC1 inhibition rescues neuronal and glial defects in NEX-*Tsc2* mutant mice

**Figure 7.**
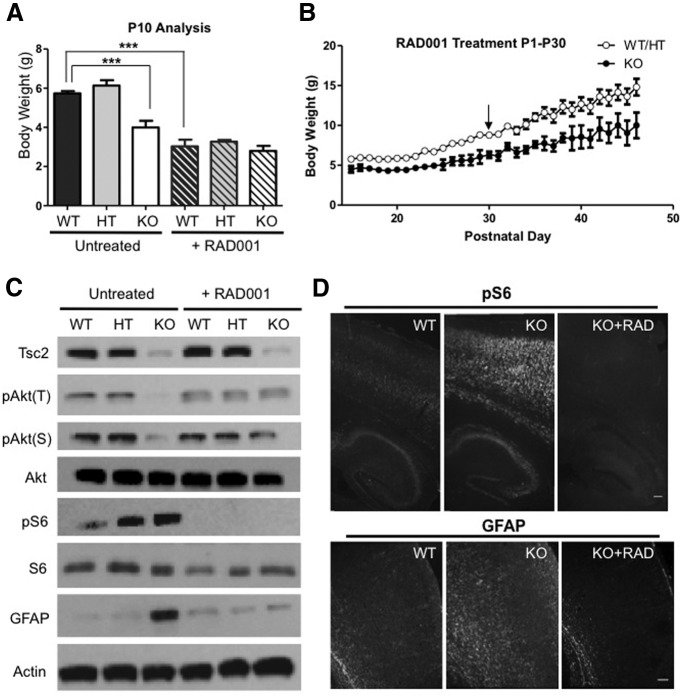
mTORC1 inhibition rescues the NEX-*Tsc2* KO phenotype. A cohort of NEX-*Tsc2* mice were treated with RAD001 daily from P1 to P9, killed at P10, and compared with an untreated cohort of the same age. ***A***, RAD001 treatment reduces the body weight of mice with all genotypes, but abolishes the growth deficit observed in the untreated cohort between the KO and WT mice. Bar graphs show the mean values ±SEM; ***p < 0.001. ***B***, Body weight plot of a different cohort of NEX-*Tsc2* littermates that were treated with RAD001 daily from P1 to P30, and measured up to P46. Data from WT (*n* = 1) and HT (*n* = 3) mice were pooled. KO mice (*n* = 2) were smaller than controls, but appeared to be healthy. ***C***, Western blot analysis of cortical lysates at P10. RAD001 treatment rescues all signaling defects, including the reduction in Akt phosphorylation and the increase in S6 phosphorylation, as well as the GFAP overexpression seen in untreated KO mice. ***D***, Immunofluorescence analysis of pS6 and GFAP expression in untreated and RAD001-treated KO mice. RAD001 treatment suppresses all staining intensity abnormalities seen in untreated KO mice. Scale bars, 200 µm.

Many previous studies (Ehninger et al., 2008; Meikle et al., 2008; Zeng et al., 2008; Ljungberg et al., 2009; Carson et al., 2012; Kazdoba et al., 2012; Way et al., 2012) demonstrated that mTORC1 inhibition *in vivo* with rapamycin or the rapalog RAD001 rescues survival and many aspects of the neuropathology of *Tsc1*, *Tsc2*, as well as *Pten* mutant mice. To investigate whether this pharmacological strategy can correct cell-autonomous neuronal defects as well as non-cell-autonomous defects in glial cells that do not exhibit mTORC1 activation, we treated a cohort of WT, HT, and KO NEX-*Tsc2* mice daily with RAD001 starting from P1, then weighted and killed all mice at P10 for analysis (Table 2). The drug treatment affected the growth of all mice, resulting in a significant reduction in the body weight of WT and HT mice compared with the untreated cohort (Fig. 7*A*). Despite their smaller size, RAD001-treated WT and HT mice appeared otherwise healthy. Untreated KO mice were significantly smaller than controls; however, the RAD001 treatment abolished this difference (Kruskal–Wallis test, *p* < 0.0001^u^; Fig. 7*A*). Strikingly, drug-treated mutant mice appeared healthy and indistinguishable from control littermates, unlike untreated mutants, which were obviously runt by P10. To examine the long-term effects of the drug, we also continued to treat one litter of NEX-*Tsc2* mice (including one WT, three HT, and two KO mice) with RAD001 daily for 30 d by monitoring their body weight. The data indicate that, although KO mice were smaller than controls, all drug-treated mice appeared healthy and thrived even after cessation of the treatment (Fig. 7*B*, Movies 1–3). Long-term RAD001-treated KO mice survived and gained weight for at least 46 days, the last time point of monitoring. However, both mice died suddenly prior to P60. Thus, RAD001 dramatically improved the well-being of KO mice and significantly extended their life span, although the beneficial effect lasted only 3-4 weeks after drug treatment cessation. In the cohort killed at P10 for analysis, we found that the drug treatment also completely suppressed the signaling abnormalities of KO mice, including the decrease in Akt phosphorylation and the increase in S6 phosphorylation (Fig. 7*C*,*D*). Levels of pAkt(T) and pAkt(S) in RAD001-treated KO mice were similar to untreated or treated controls. Levels of pS6, however, were virtually undetectable in all drug-treated mice, demonstrating the effectiveness of the treatment and the expected, powerful inhibition of mTORC1 signaling (Fig. 7*C*,*D*). Furthermore, the treatment strongly suppressed glial abnormalities in KO mice (Fig. 7*C*,*D*). GFAP levels in RAD001-treated mutants were similar to those in controls by either Western blot or immunofluorescence analysis (Fig. 7*C*,*D*). These data demonstrate that the suppression of elevated mTORC1 activity in postmitotic *Tsc2* mutant neurons is sufficient to prevent defective neuroglia communication signals that promote glial abnormalities in the developing postnatal neocortex.

## Discussion

In this study, we describe a novel conditional *Tsc2* mouse mutant line in which deletion of the gene occurs specifically in excitatory neurons of the developing forebrain. Because these neurons are the most abundant cell type in the developing neocortex, we reasoned that they may drive the pathology of *Tsc2* mutant mice through cell-autonomous as well as non-cell-autonomous mechanisms. Indeed, we found that homozygous NEX-*Tsc2* mice exhibit neuronal as well as glial abnormalities, even though *Tsc2* gene deletion and mTORC1 signal activation were present only in excitatory neurons. Furthermore, we found that treatment with the mTORC1 inhibitor RAD001 strongly suppressed the phenotype of mutant mice by rescuing neuronal as well as glial abnormalities. These findings suggest that *Tsc2* mutant excitatory neurons produce mTORC1-dependent extracellular signals that can influence the development or proliferation of genetically normal astrocytes in the neocortex. Future studies will be required to identify the abnormal neuron–glia communication signals in NEX-*Tsc2* mutant mice. These studies will offer new insights into basic mechanisms of brain development, and may lead to the identification of new drug targets for the prevention and treatment of astrogliosis in TSC. Given the contribution of reactive astrocytes to the etiology of epilepsy, treatments that effectively suppress astroglia dysfunction, particularly in the developing brain, may be beneficial to patients that are affected by an mTORC1-related pathology, including TSC, cortical dysplasia, or traumatic brain injury (Wong and Crino, 2012; Lipton and Sahin, 2014).

Like previous mouse models in which *Tsc2* was deleted specifically in neural progenitors, astrocytes, or neurons, our excitatory neuron-specific NEX-*Tsc2* homozygous mice exhibited premature death at early postnatal ages and responded very well to postnatal treatment with the rapamycin analog RAD001. The cause of death of homozygous mutants is not presently known, but it is likely due to muscle wasting and failure to thrive given the runt appearance and ataxic behavior of the mutants. In contrast, RAD001-treated mutants thrived both during and after weaning despite their small size. The suppression of the mutant phenotype resulting from the RAD001 treatment was very remarkable, and mutant mice could hardly be distinguished from drug-treated control mice based on their general appearance (Movies 1–3). The extended drug treatment did cause hair loss in all drug-treated mice, particularly in the single cohort treated for 30 d; however, hair mostly regrew within 1–2 weeks after treatment cessation. Mutant mice continued to survive and appeared healthy at least until 46 days, the last time point of observation. During this time, we did not observe overt seizure activity in treated or untreated NEX-*Tsc2* mutant mice. However, further investigation with video-EEG recordings will be required to ascertain whether or not these mice have normal baseline activity or experience spontaneous subclinical seizures, or have decreased seizure threshold after exposure to chemoconvulsant agents.

As previously reported in neuron-specific *Tsc1* mutants (Meikle et al., 2007), we observed a subtle, but noticeable, intrinsic neuronal phenotype in our NEX-*Tsc2* homozygous mutants. Overall brain size and weight were not changed (Fig. 2, Table 2), but the neuronal soma and the extension of cellular processes did appear increased, especially when dissociated cells were grown in culture (Fig. 6). There was a mild dyslamination of the deep cortical plate and the hippocampal pyramidal layer in area CA1, and a modest cellular ectopia near the cortical subplate region (Fig. 2). However, most mutant principal neurons, particularly those destined to upper cortical layers (Cux1-positive), occupied the appropriate cellular positioning in forebrain structures, suggesting that radial migration is not strongly affected by the loss of *Tsc2* or the upregulation of mTORC1 signaling. Our findings contrast with those of a previous study (Tsai et al., 2014), in which *Tsc2* knock-down neurons were shown to fail to migrate to appropriate upper cortical layers and, furthermore, disrupted the migration of surrounding untransfected Cux1-positive neurons. However, it should be noted that the knockdown by *in utero* electroporation approach used in that study primarily targeted neural progenitor cells and their progeny destined for upper cortical layers, and clearly affected the acquisition of neuronal identity, since most knock-down neurons failed to express neuron markers characteristic of either upper or deep cortical layers. In contrast, our gene knockout approach targeted specifically postmitotic cortical neurons destined to all cortical layers, but preferentially affected the positioning of deep layer neurons (Tbr1-positive). Further birth-dating and marker analysis will be required to fully understand the developmental origin of the cellular ectopia in NEX-*Tsc2* mutants.

The neuroanatomical phenotype of our NEX-*Tsc2* mutants was relatively mild compared with other *Tsc1* or *Tsc2* conditional knock-out models, particularly those driven by promoters that are active in radial progenitor cells, such as Emx1 (Magri et al., 2011; Carson et al., 2012), nestin (Goto et al., 2011), or human GFAP (Way et al., 2009). This is most likely due to the cell type-specific nature of the knockout (postmitotic, excitatory neuron restricted in the case of NEX-*Tsc2* mutants) rather than the developmental time of onset of gene deletion, which occurs at similar times in these mutants during embryonic corticogenesis (Goebbels et al., 2005; Belvindrah et al., 2007). We interpret these findings to suggest that most excitatory cortical neurons do not require Tsc2 in order to migrate radially into the cortical plate, although this activity is needed for optimal positioning of early-born neurons in deep cortical layers. The loss of *Tsc1* or *Tsc2* in radial progenitor cells of other mutants may be more devastating because it disrupts the radial scaffold or affects the identity of the neuronal progeny. However, we cannot presently rule out the possibility that the milder migration phenotype of our mutants may be due to a delay in the loss of the Tsc2 protein in excitatory cortical neurons due to protein stability.

We observed non-cell-autonomous astrogliosis in the NEX-*Tsc2* homozygous mutant neocortex. Our findings are consistent with the fact that astrogliosis is commonly observed in human patients afflicted by TSC or other forms of epilepsy (Wong and Crino, 2012). However, astrogliosis was not observed in previous studies using the synapsin I promoter to drive neuron-specific conditional deletion of *Tsc1* (Meikle et al., 2007), or when *Tsc1* was deleted in isolated neurons using the *in utero* electroporation technique (Feliciano et al., 2011). The reason for this discrepancy is not presently clear. It is possible that the neuron-specific deletion of *Tsc2* is more deleterious than that of *Tsc1*, as has been suggested for astrocyte-specific gene knock-out mice (Zeng et al., 2011). It is conceivable that *Tsc2*, but not *Tsc1* mutant neurons significantly alter neuroglia communication in the developing neocortex, possibly by altering the levels of a secreted molecule that accumulates in the extracellular environment. It is interesting to note that astrogliosis in NEX-*Tsc2* mutants was most pronounced in deep cortical layers, in close physical association with neuronal abnormalities. Other possibilities are that astrogliosis in our mutants is a secondary result of cell death or seizure activity. The possibility that astrogliosis in NEX-*Tsc2* KO mice results from cell death was ruled out in the present study. However, the possible role of seizures was not directly addressed and at present cannot be ruled out. Even though seizures were not apparent in our mice, whereas they were clearly present in synapsin I-*Tsc1* mice in the absence of astrogliosis (Meikle et al., 2007), it is conceivable that abnormal electroencephalographic activity could induce astrocyte alterations. Further studies are required in the future to directly address this issue. Further studies will also be needed to determine whether *Tsc2* mutant neurons disrupt the proliferation, migration, or survival of cortical astrocytes. In addition to astrocytes, we also attempted to examine the status of other glial cells using antibody markers such as Iba1 for microglia and Olig2 for oligodendrocytes. However, sample variability among P10 mice was very high, and no consistent genotype-dependent effects were seen (data not shown). Given the early mortality of our mutants, this issue will have to be addressed in the future by culture studies.

Video 1. RAD001-treated NEX-*Tsc2* WT mouse at P15. 10.1523/ENEURO.0046-15.2015.video.1

Video 2. RAD001-treated NEX-*Tsc2* KO mouse at P15. 10.1523/ENEURO.0046-15.2015.video.2

Video 3. Untreated NEX-*Tsc2* KO mouse at P15. 10.1523/ENEURO.0046-15.2015.video.3
